# Identifying patient-related predictors of permanent growth hormone deficiency

**DOI:** 10.3389/fendo.2023.1270845

**Published:** 2023-10-10

**Authors:** Veronica Mericq, German Iñiguez, Graziella Pinto, Laura G. Gonzalez-Briceño, Dinane Samara-Boustani, Caroline Thalassinos, Isabelle Flechtner, Athanasia Stoupa, Jacques Beltrand, Alix Besançon, Séverine Brabant, Khaldoun Ghazal, Monique Leban, Philippe Touraine, Gabriel Cavada, Michel Polak, Dulanjalee Kariyawasam

**Affiliations:** ^1^Institute of Maternal and Child Research, School of Medicine, University of Chile, Santiago, Chile; ^2^Paediatric Endocrinology, Diabetology, and Gynaecology Department, Necker-Enfants Malades University Hospital, Assistance Publique-Hôpitaux de Paris, Paris, France; ^3^Institut Imagine, Paris, France; ^4^Université Paris Cité, Paris, France; ^5^Functional Exploration Department, Necker-Enfants Malades University Hospital, Assistance Publique-Hôpitaux de Paris, Paris, France; ^6^Endocrinology Department, La Pitié-Salpêtrière University Hospital, Paris, France; ^7^Public Health Department, Faculty of medicine, University of Chile, Santiago, Chile

**Keywords:** growth hormone deficiency, transitional period, care transition, growth hormone testing, permanent GHD predictors

## Abstract

**Objective:**

Isolated childhood growth hormone deficiency (GHD) can persist into adulthood, and re-testing at the transition period is needed to determine whether continued growth hormone therapy is indicated. Here, our objective was to identify predictors of permanent GHD.

**Design:**

Retrospective single-centre study of patients with childhood-onset GHD who were re-tested after adult height attainment.

**Methods:**

Auxological, clinical, laboratory, and MRI data throughout follow-up were collected.

**Results:**

We included 101 patients. At GH treatment initiation, age was 8.1 ± 0.4 years, height -2.25 ± 0.8, and BMI -0.27 ± 0.1 SDS. The 29 (28.7%) patients with persistent GHD had lower height SDS (-2.57 ± 0.1 vs. -2.11 ± 0.1, *p*<0.001) and mean GH peaks (8.4 ± 1.0 vs.13.2 ± 0.5 mIU/L, *p*<0.001) at GHD diagnosis; at adult height, they had lower IGF1 (232 ± 19.9 vs. 331 ± 9.1 ng/mL, *p*<0.001) and higher BMI SDS (-0.15 ± 0.27 vs. -0.73 ± 0.13, *p*<0.005). By multivariate analysis, the best predictive model included height and BMI SDS, both GH peaks, and MRI findings at diagnosis. Patients with height at diagnosis <-3 SDS had a 7.7 (95% IC 1.4-43.1, p=0.02) fold higher risk of persistent GHD after adjustment on BMI SDS. An abnormal pituitary region by MRI was the strongest single predictor (7.2 times, 95% CI 2.7-19.8) and after multivariate analysis adjustment for GH peaks and height SDS at diagnosis, the risk increased to 10.6 (1.8 - 61.3) times.

**Conclusions:**

Height <-3 SDS at GHD diagnosis and pituitary MRI abnormalities should lead to a high index of suspicion for persistent GHD.

## Introduction

Growth hormone deficiency (GHD) is the most common endocrine disorder in children with short stature, affecting about one in 4000 (1). The diagnosis rests on a combination of auxological, biochemical, and neuroradiological data. Given the pulsatile pattern of growth hormone (GH) secretion, provocative testing must be performed. The results are difficult to interpret, however, notably due to the considerable intra- and inter-subject variability in GH production and to uncertainty about the optimal cut-offs for defining GHD. GH provocative tests lack of reproducibility and have a high rate of false-positive results (2, 3). This fact leads us to the the question of whether this represents a form of truly transient GHD or a false positive diagnosis during childhood. On the other hand, GH secretion is considered a continuum from normality to severe GHD with large intra and inter subject variability. Based on these facts, the latest guidelines suggest that the cut-offs of the GH peak for GHD should be reduced in the attempt to minimize the large number of false-positive results (4).

In patients with childhood-onset GHD, GH replacement is typically discontinued once linear growth is complete. Patients are then re-tested and usually have normal stimulated GH responses (5, 6). Identifying patients with persistent GHD requiring further GH therapy is crucial, as GH not only stimulates linear growth but also exerts beneficial effects on body composition, peak bone mass achievement (7), metabolic and cardiovascular health, and quality of life (8). Maintaining optimal replacement during the transition from late adolescence to adulthood, i.e., 6–7 years after linear growth completion, is particularly important to optimize lifelong outcomes (9). Patients with GHD can be categorized as at high, moderate, or low probability of persistent GHD based on clinical and biochemical variables (10). Knowledge of the factors that predict childhood-onset GHD persistence into adulthood would be valuable to identify patients warranting a high index of suspicion of persistent GHD during the transition period.

The objective of this retrospective single-centre cohort study was to identify predictors of permanent GHD in patients with a childhood diagnosis of non-tumor-related isolated GHD.

## Methods

This study was approved by the Necker-Enfants Malades University Hospital (Assistance Publique-Hôpitaux de Paris) ethics committee (N° 2023 0202122136), which waived the need for patient informed consent in compliance with French law on retrospective studies of de-identified healthcare data.

We performed a retrospective single-centre observational cohort study in patients diagnosed with GHD at the Paediatric Endocrinology Department of the Necker-Enfants Malades university hospital (Paris, France) then followed-up between 1 January 1993 and 31 August 2021 until adult height was achieved and patients re-tested.

### Diagnosis and management of childhood-onset growth hormone deficiency

GHD was suspected in patients whose height was below –2 SDS or at least 1.5 SDS below mid-parental height or growth velocity decreased after infancy by at least 0.3 SDS/year from their initial height SDS. In neonates and toddlers, GHD was suspected if hypoglycaemic episodes occurred.

**Figure 1:**
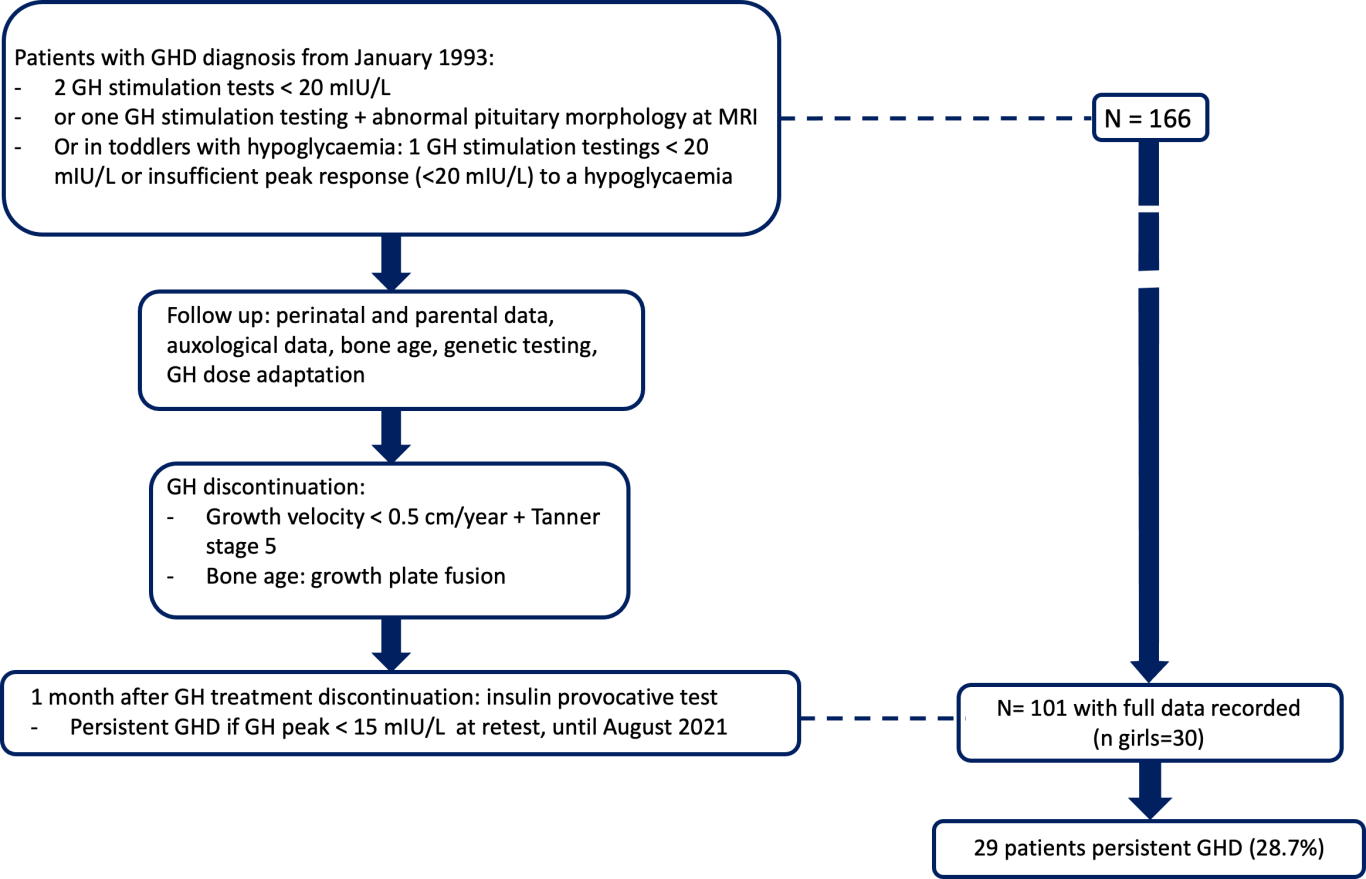
Study flow chart.

Confirmed GHD was defined as GH peak responses to two provocative tests below 20 mIU/L; however, in patients with abnormal pituitary morphology by magnetic resonance imaging (MRI) or with hypoglycaemic episodes, an inadequate response below 20 mIU/mL to a single test was sufficient (**Figure 1**). GH stimulation was with glucagon in patients weighing less than 15 kg and with combined arginine and insulin in patients weighing 15 kg or more. Normal IGF1 values (≥ - 2 standard deviation score [SDS] for age and pubertal stage) did not exclude GHD. We excluded patients who had unconfirmed GHD, refused study participation, had missing data, fusion of growth plates or had pituitary tumors (adenoma, craniopharyngioma, or glioma), patients with a syndromic presentation, chromosomal abnormalities, skeletal dysplasia, chronic disease with an impact on growth (ie. chronic inflammatory disease, chronic kidney disease), patients receiving chronic treatment that could influence growth (ie. glucocorticoids). Pubertal patients were not excluded.

Once GHD was diagnosed as described above, GH therapy was given until adult height was achieved, i.e., height velocity fell below 0.5 cm/year in a patient who was Tanner stage 5 or had growth-plate fusion with adult bone age on radiographs (15 years in girls and 17 years in boys). According to guidelines, GH treatment was administered at a dosage between 0.025 and 0.035 mg/kg/d. An insulin GH provocative test was performed at least one month after GH treatment discontinuation. Permanent GHD was defined as a GH peak <15 mIU/L in response to this test.

### Data collection

We collected the following information from the medical records: auxological data during the perinatal period, at GHD diagnosis, and during follow-up; hormone assay results and other laboratory test results; pubertal data (Tanner stage, clinical evaluation of testicle size); mid-parental height ((maternal height + paternal height + 13)/2 for a boy or (maternal height + paternal height – 13)/2 for a girl); bone age assessed according to Greulich and Pyle; genetic test results; and MRI findings. Auxological data were reported using French growth charts (11).

### Hormone assay methods

GH was measured using the DXI Beckmann Coulter assay with a detection limit of 0.03 mIU/L and inter- and intra-assay detection limits of 3.7%–4.0% and 4.6%–5.7%, respectively.

IGF-1 levels were measured by the Cis Bio International immunoradiometric assay until 1 August 2013 and the IDS-iSYS IGF1 assay subsequently and were interpreted using reference values according to age and pubertal status. IGF1 levels are dependent on the nutritional status and can be normal in obese/overweight patients with a GH deficiency or can be decreased in undernourished patients, without any GH deficiency. Thus, IGF1 levels were not considered to define permanent GHD. Patients with insufficient GH peak at the final GH stimulation were considered permanent GHD even when IGF1 levels were normal.

Free thyroxine (FT4) and thyroid-stimulating hormone (TSH) were measured using the DXl Beckmann Coulter assay; follicle-stimulating hormone (FSH) using the Cobas Roche immunoassay with inter- and intra-assay detection limits of 2.5%–2.7% and 1.4%–1.7%, respectively; and luteinizing hormone (LH) using the Cobas Roche immunoradiometric assay with inter- and intra-assay detection limits of 2%–2.4% and 0.8%–2.9%, respectively.

#### Magnetic resonance imaging (MRI)

Hypothalamic and pituitary MRI was performed in all patients with a confirmed GH deficiency as part of the work up for every child who has clinical and biochemical diagnosis of GHD in order to rule out anatomical defects. MRI was considered abnormal if one or more congenital abnormalities were found in this anatomic region: ectopic posterior pituitary, thin or interrupted pituitary stalk, absence of spontaneous signal of posterior pituitary, small anterior pituitary (height less than 2.5 cm), Rahtke pouch cyst. Patients with septo-optic dysplasia, holoprosencephalia or other brain malformations were excluded.

### Statistical analysis

Descriptive anthropometric and hormonal characteristics were described as median with interquartile ranges for not-normally distributed continuous variables and as frequency and percent for categorical variables.

The compared selected study variables between children with vs. without persistent GHD were gestational age, birth weight, birth length and head circumference, age at first consultation, age at initiation of GH, bone age at GH initiation, baseline IGF 1 and peak of GH at two GH provocative test, mean of the peak of the two GH provocative tests, age at menarche in girls, height, weight and BMI SDS at GH initiation, age reaching a stage Tanner 2, parental height, height, weight, BMI SDS, IGF-1 and GH peak at adult height. We used the Mann Whitney test for this comparison. To identify predictors of persistent GHD (dependent variable), we used a logistic regression model with explanatory variables: height and BMI SDS at diagnosis and birth weight SDS, hormonal (both GH peak and IGF-1 at diagnosis) and MRI variables (normal or abnormal hypothalamic- pituitary region). Next, we built a multivariate model using the variables associated with *p* values <0.05 by univariate analysis, using stepwise selection with retention ´probability equal to 0.05.

For quantitative predictors of persistent GHD, we plotted the receiver operating characteristic (ROC) curve and identified the best cut-off (i.e., the value associated with the highest Youden index) for predicting persistent GHD.

All confidence intervals were level 95%, and we used a statistical significance of 0.05. Data were processed in statistical software STATA v.17.0.

## Results

Of 166 patients seen at our department for GHD and having attained their adult height during the study period, 101 had complete information (more than 90% of the selected data set), 71 males and 30 females (**Figure 1**). **Table 1** reports their main features (Means ± SE). Adult height minus target height was -0.32 SDS (IQ25 -0.87; IQ75 0.30), and when retested after GH therapy completion, mean IGF-1 was 313.0 ng/mL (IQ25 248.9; IQ75 370.8), and GH peak was 23.7 mIU/L (IQ25 11.6; IQ75 39.0).

**Table 1:** Main features of the 101 study patients (median, IQ25 – IQ75).

	ALL (n=101), median (IQ25; IQ75)	Non persitent GHD (n=72), median (IQ25 IQ75)	Persistent GHD (n=29), median (IQ25; IQ75)	p
**Gestational age (weeks)**	39.0 (38.0; 40.0)	39.0 (38.0 40.0)	39.0 (38.8 40.0)	0.229
**Birth weight (percentile)**	39.5 (14.8;663)	31.5 (13.3; 58.6)	47,4 (30.1; 78.0)	0.102
**Birth length (percentile)**	37.6 (7.8;54.6)	31.0 (5.9; 66.2)	42.6 (31.0; 57.8)	0.352
**Head circumference (Percentile)**	46.2 (23.3;71.0)	45.6 (24.7; 66.0)	48.1 (28.9; 75.4)	0.745
**SGA number (%)**	24.8	28.2	17.2	**0.001**
**Age at first visit (years)**	8.9 (5.4;11.6)	8.3 (5.5;106)	10.3 (4.5; 13.8)	0.345
**Bone age at GH start (years)**	8.0 (5.0;11.0)	7.0 (4.0;10.5)	(2.7;12.3)	0.886
**Chron. age-Bone age (years)**	23 (1.5;3.4)	2.3 (1.2; 3.4)	1.8 (1.4; 2.5)	0.510
**First IGF-1 ng/ml**	92.0 (64.0;139.0)	92.0 (65.0;211.6)	87.0 (43.0;125.0)	**0.049**
**First GH stim. Peak mUI/L**	11.1 (8.0 16.0)	12.2 (8.5 19.0)	7.8 (4.5; 18.0)	**0.001**
**Second IGF-1 (ng/ml)**	96.5 (63.5;150.5)	96.0 (70.5 168.0)	113.0 (27.5, 199.3)	0.977
**Second GH stim. Peak (mUI/L)**	12.0 (8.1;15.9)	12.8 (9.5; 17.0)	7.1 (3.8;13.3)	**0.001**
**Mean 1 + 2 GH Stim.peak (mUI/L)**	11.8 (8.8; 15.1)	13.2 (10.5 15.4)	7.9 (4.4;11.9)	**0.001**
**Mean IGF1 1 + 2 baseline (ng/ml)**	101.8 (69.0; 144.6)	98.0 (73.5; 152.0)	109.5 (49.3; 183.8)	NS
**Age at GH start (years)**	9.8 (8.8;15.1)	10.2 (6.5; 12.3)	10.1 (4.0; 13.3)	0.867
**Height at GH start (SDS)**	-2.34 (-2.78; -1.98)	-2.17 (-2.56; -1.77)	-2.66 (-2.97; -2.11)	**0.012**
**BMI at GH start (SDS)**	-0.32 (-1.00 -0.29)	-0.29 (-1.00; 0.11)	0.08 (-0.44 0.82)	**0.032**
**Age at Tanner2 (years) girls**	11.6 (10.1; 12.6)	11.6 (10-4; 12.7)	11.3 (9.9; 12.8)	NS
**Age at Tanner2 (years) boys**	13.5 (12.6 14.5)	13.3 (10.4 13.9)	13.7 (12.2 14.2)	NS
**Age at menarche (years)**	13.7 (12.8 14.6)	13.9 (12.9; 14.8)	12.9 (12.2 14.9)	0.461
**IGF-1 after GH stop (ng/ml)**	313.0 (248.9;37	324.0 (269.0;388.0)	224 (140.3; 311.5)	**0.001**
**GH stim. peak after GH stop mUI/L**	23.7 (11.6;39.0)	30.4 (21.4;41.3)	8.1 (4.4;11.0)	**0.001**
**Final height (SDS)**	-0.85 (-1.40 -0.36)	-0.63 (-1.25; -0.30)	-0.79 (-1.54 -0.40)	0.472
**Final weight (SDS)**	-0.86 (-1.63 -0.13)	-0.89 (-1.41; -0.24)	-0.31 (-0.94 0.22)	**0.020**
**Final BMI (SDS)**	-0.40 (-1.56 0.21)	-0.59 (-1.37; 0.00)	0.19 (-0.63 0.54)	**0.006**
**Mother height (SDS)**	-0.17 (-0.90 0.29)	-0.25 (-0.90; 0.29)	-0.02 (-0.90; 0.44)	0.830
**Father height (SDS)**	-0.30 (-0.85; 0.30)	-0.30 (-0.85; 0.44)	-0.30 (-0.85; 0.23)	0.951
**Target height (SDS)**	-0.32 (-0.87; 0.15)	-0,30 (-0.70 0.24)	-0.33 (-1.10 0.10)	0.305

Bold values: p < 0.05 (significant values). NS, non significant.

After adult height attainment, 29 (28.7%) patients had persistent GHD. **Table 1** compares them to the patients with transient GHD. The persistent GHD group had fewer patients born small for gestational age; higher body mass index (BMI), lower IGF-1, and lower mean GH peak at GHD diagnosis; shorter height at GH initiation; a higher BMI (0.08 (IQ25 -0.44; IQ75 0.82) versus -0.29 SDS (IQ25 -1.00; IQ75 0.11, p=0.032) and a lower mean GH peak at diagnosis (7.9 mIU/L (IQ25 4.4; IQ75 11.9) versus 13.2mIU/L (IQ25 -10.5; IQ75 15.4, p=0.001), and lower IGF-1 (224 (IQ25 140.3; IQ75 311.5) versus 324.0 (IQ25 269.0; IQ75 388.0 ng/ml, p=0.001) and higher BMI (0.19 (IQ25 -0.63; IQ75 0.54) versus -0.59 (IQ25 -1.37; IQ75 0.00) SDS, p=0.006) at adult height. Three patients with persistent GHD had gene mutations associated with abnormal pituitary development (*GLI2* in two patients and *PROP1* in one patient).

Ten children were in puberty when initially evaluated (8 boys and 2 girls). No significant difference was found in their response to the first and second stimulation test (**Supplementary Figure 1**) when compared to pre-pubertal children.

Of the 29 permanent GHD, there were 20 boys and 9 girls. Girls with permanent GHD had a significant lower final height compared to boys with permanent GHD (-1.25 SDS vs -0.58 SDS, p= 0.0024), whereas no difference was observed in the age and height at treatment initiation, GH peaks after stimulation, pituitary abnormalities. As puberty onset occurred in normal ranges and the age at treatment initiation was similar, we suspect that the girls had less time to catch up their stunted growth (**Supplementary Figure 2**).

### Magnetic resonance imaging findings

Of the 29 patients with persistent GHD, 17 (58.6%) had MRI abnormalities of the pituitary region. The most common abnormality was an ectopic posterior pituitary with a small anterior pituitary and a thin or interrupted pituitary stalk (7/29, 24.1%). The other abnormalities were a small anterior pituitary (5/29, 17.2%), a thin pituitary stalk (3/29, 10.3%), and a Rathke pouch cyst (1/29, 3.4%).

Of the 72 patients with transient GHD, 14 (23.6%) had MRI abnormalities in the pituitary region: 12 had a small anterior pituitary (12/72, 16.7%) and two an ectopic posterior pituitary with a small anterior pituitary (2/72, 2.8%).

### Other pituitary-hormone deficiencies

All patients had thyroid hormones assessments before GH therapy. Of the 29 patients with persistent GHD, five developed central hypothyroidism (17.2%) compared with seven of the 72 patients with transient GHD (9.7%) (p<0.01); none of these cases were transient central hypothyroidism (*p*<0.01). In the transient GHD group, one patient developed over time both central hypothyroidism and pubertal delay and another developed precocious puberty.

Regarding the five patients with hypothyroidism among the 29 patients with persistent GHD, all had a central hypothyroidism. For two of them, central hypothyroidism was diagnosed at the same time of GH deficiency, was persistent during one year after GH discontinuation for one patient and still under treatment for central hypothyroidism for the other one while GH therapy was discontinued. For the other three of them, central hypothyroidism was diagnosed at least one year after GH deficiency diagnosed. Of these three patients, two had persistent hypothyroidism whether GH therapy was discontinued or not and one had a transient hypothyroidism during puberty and Levothyroxine treatment was discontinued when GH therapy was discontinued. These central hypothyroidisms are therefore probably due to an extensive pituitary insufficiency.

### Predictors of persistent growth-hormone deficiency

In an univariate analysis persistent GHD was best predicted at diagnosis by a shorter height SDS (<-3.0 SDS) OR=7.7 (1.36 – 43.1, p=0.021), a lower mean GH peak OR=0.85 (0.77– 0.94, p=0.001), an abnormal MRI OR=7.2 (2.65-19.76, p=0.001) and a higher BMI SDS OR=7.2 (2.65-19.76, p=0.044).

In the multivariate analysis (**Table 2**) height (< - 3 SDS) loses statistical significance, possibly due to a large confidence interval, however its positive association with the permanent GHD condition is still suggestive. Instead, a large GH peak is a significant protection against permanent GHD. For each unit increase of GH peak there is a 20% reduction in the chance of permanent GHD. An abnormal pituitary region by MRI was the strongest single predictor, increasing the risk of developing permanent GHD twelve fold. Similarly an increase of 1 unit of BMI, increases the risk of permanent GHD by 2.3 times.

**Table 2:** Multivariate analysis of predicting factors.

GHD adult	Odds Ratio	SE	z	p	[95% CI]
**Height**	5.617209	6.137018	1.58	0.114	[0.6600091 – 47.80697]
**GH peak**	0.8034541	0.0648711	-2.71	0.007	[0.685859 - 0.9412116
**MRI altered**	12.46626	9.620868	3.27	0.001	[2.746749 – 56.57873]
**BMI sds**	2.266474	0.8812106	2.10	0.035	[1.057795 - 4.856238]
**cons**	1.069849	0.8423922	0.09	0.932	[0.228608 – 5.006719]

**Figure 2:**
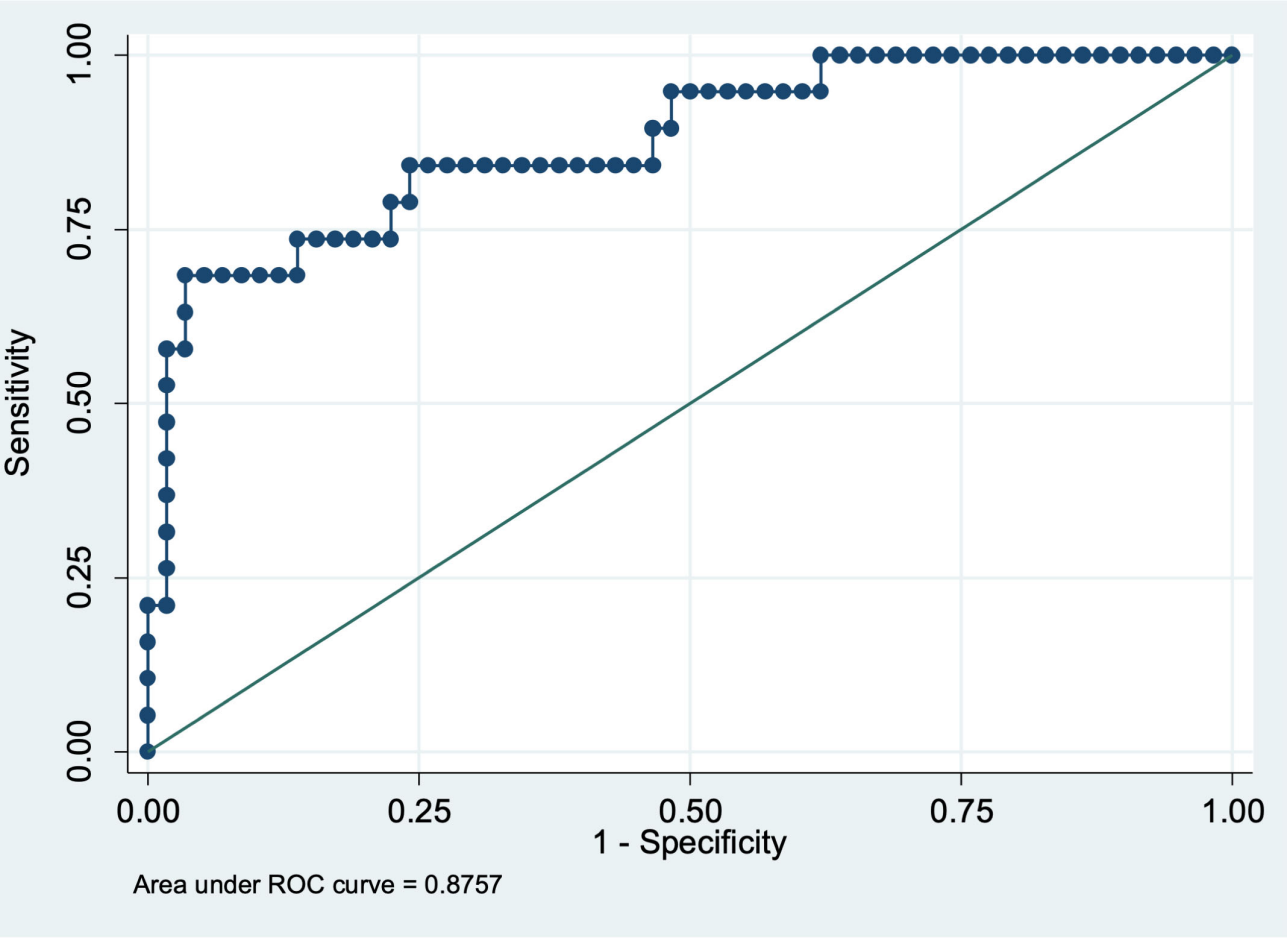
ROC curve for estimation of sensitivity and specificity of the model.

This model has a prognostic discrimination capacity of 87.6% estimated through AUC, 77.6% specificity and 73.7% sensitivity (**Figure 2**).

## Discussion

In our study, most patients with non-tumoral isolated GHD diagnosed in childhood no longer had GHD at linear growth completion, in keeping with previous data (12, 13). Predictors of persistent GHD available at the diagnosis of GHD were shorter height, higher BMI, lower post-stimulation GH peaks, and presence of pituitary abnormalities by MRI. Height <-2.5 SDS at GHD diagnosis had high sensitivity, specificity, and positive predictive value for persistent GHD. Thus, children who have a very short stature (<-3 SDS) at GHD diagnosis with no other identifiable cause (e.g., skeletal dysplasia) can be considered very likely to have persistent GHD. The strongest single predictor of persistent GHD was the presence of pituitary MRI abnormalities.

The classical presentation of childhood GHD is short stature, frontal bossing, mid-facial hypoplasia (doll-like facies), and truncal adiposity. During infancy, hypoglycaemic episodes, prolonged jaundice and micropenis may occur and should be considered as an indication of congenital GHD. However, these features are inconsistent and not specific (ie micropenis can also be due to a congenital hypogonadotropic hypogonadism), and diminished growth velocity, even in the absence of short stature, should lead to diagnostic tests for GHD. Outside the neonatal period, random GH measurements are not reliable for GHD diagnosing (14). After excluding other causes of slow growth, GH provocative testing must therefore be performed (4). However, the results are difficult to interpret, and recommendations have been made on this point (15) (16),. The Growth Hormone Research Society workshop concluded that, with the currently available tests, peak values ≤20 mIU/L (<6.7 ng/mL) in response to two different secretagogues are required for the diagnosis of GHD in most children. However, when the index of suspicion is high, a single test may be sufficient.

In our study, a lower GH peak at diagnosis was associated with persistent GHD, consistent with an earlier report (17). That this was not the best predictor is in accordance with the poor reproducibility of GH provocative tests and high proportion of false-positive results with failure to increase GH secretion even in healthy children (18). In our study, the criteria for GH provocative test positivity were those indicated in guidelines (15) and the proportion of patients with persistent GHD was similar to that in a recent study that used similar criteria (17). In the past, a 12-h or 24-h overnight GH profile with blood sampling every 20 minutes was used to diagnose GHD. Compared to provocative tests, this method is more reproducible (19–22), but less sensitive, failing to diagnose 57% of cases identified by GH provocative tests (20). Consensus guidelines issued in 2016 (15) and 2019 (10) indicate that GH profile results should not be used to identify patients requiring GH therapy.

The mechanisms of GH secretion recovery in patients with transient childhood GHD are unclear. One possibility is that hypothalamic-pituitary function, notably GH secretion, improves after puberty. However, the recovery may be only apparent, with secretory deficiency being initially misdiagnosed due to a false-positive provocative test in a patient with short stature or pubertal delay. Another possibility is modification of the criteria for GH provocative test positivity between the diagnosis of GHD and re-testing after adult height attainment, as GH need differs across time (lower needs after puberty). The type of test used, age, BMI, disease duration, number of pituitary hormone deficiencies, and presence of pituitary abnormalities may also play a role in test results (9). Spontaneous GH secretion increases during pubertal development and is thus much higher in adolescents than in adults (23–25). In one study, 55% of children with isolated GHD already had sufficient GH secretion at mid-puberty (26). During childhood, patients with all degrees of GHD (peak GH <20 mIU/L) receive GH therapy, whereas at the transition to adulthood and in adulthood the lower cut-offs of 15 and 10 mIU/L, respectively, are used; thus, for re-testing in our patients after adult height achievement, we used the 15 mIU/L cut-off. In addition, even in patients likely to have impaired GH secretion due, for instance to congenital hypothalamic-pituitary abnormalities or radiation therapy to the pituitary in childhood, peak GH responses may change over time and must therefore be assessed repeatedly during long-term follow-up (27).

Using a lower GH-peak cut-off for diagnosing GHD may decrease the number of false-positive results but may also lead to mild forms of GHD being missed (28). Patients with mild GHD may benefit from GH therapy (17) but Rodari et al. noticed that height gain was rather poor in “mild GHD”. In our cohort, GH patients with transient GHD had a height gain, supporting the diagnosis of milder forms of GHD as opposed to GHD overdiagnosis.

Abnormal pituitary morphology other than isolated small anterior pituitary size or Rathke pouch cyst by MRI strongly predicted persistent GHD in our study. In our cohort, normalization of GH secretion occurred even in patients with small anterior pituitaries. However, all 10 patients with pituitary stalk hypoplasia had persistent GHD. Conceivably, small anterior pituitary size as the only abnormality may have limited clinical relevance and may normalize a few years later (5). In contrast, an ectopic neurohypophysis, irrespective of the appearance of the pituitary stalk, may be more likely to indicate persistent GHD (29). Nevertheless, two of our patients with an ectopic neurohypophysis had transient GHD. Similarly, a reassessment of the GH status of patients with ectopic neurohypophysis showed persistent severe GHD in only 61% of cases. Even after GH secretion recovery, patients with ectopic neurohypophysis should receive lifelong body-composition monitoring and further GH testing if abnormalities are detected (30).

Complete pituitary-stalk agenesis indicates a severe form of GHD with ectopic neurohypophysis located at the median eminence and with multiple anterior pituitary-hormone deficiencies (30).

Ectopic neurohypophysis with a visible pituitary stalk is usually located more proximally and is more often associated with GHD as the only pituitary-hormone deficiency (30). Nevertheless, pituitary function should be assessed periodically in patients with MRI pituitary abnormalities and isolated GHD, as other pituitary-hormone deficiencies may develop over time (31). In our cohort of patients with initially isolated GHD, the subsequent development of other pituitary-hormone deficiencies was uncommon and usually consisted in central hypothyroidism. In contrast, in another study, 45% of 83 patients with initially isolated GHD subsequently developed other pituitary-hormone deficiencies, after a median of 5.4 years, and FSH and LH were the hormones most often affected (32).

IGF-1 at diagnosis was not a strong predictor of GHD persistence after linear growth completion. The regulation of IGF-I secretion and effects is complex, and IGF-I serum concentrations are affected by many factors such as age, endogenous GH secretion, BMI, physical fitness, glucocorticoid exposure, prolactin and testosterone levels, and IGFBP-3 binding capacity (33). Whether serum IGF-I levels should be interpreted according to chronological age, bone age, or pubertal development is a matter of discussion. In 154 peripubertal patients with short stature, assessing IGF-1 levels based on pubertal stage had greater positive predictive value for diagnosing GHD than the assessments based on chronological age and bone age (34). However, the assessment based on chronological age was more sensitive, suggesting greater value as a screening tool. In children younger than three years of age, IGFBP-3 is considered a more reliable biomarker of GH secretion than IGF-1 (4). Only three patients in our cohort were younger than three years at GHD diagnosis and, consequently, we did not analyse this variable.

Continuing GH therapy during the transition period is important but difficult to implement in clinical practice (35). GHD is associated with osteopenia, glucose and lipid metabolism disturbances, an increased prevalence of cardiovascular disease, increased fat mass and decreased lean mass, and impaired quality of life (8, 36). Consequently, patients with persistent GHD must continue to receive GH replacement. Multiple studies suggest that GH therapy discontinuation during puberty does not have an impact on adult height (26, 37, 38), when GH therapy was started early and when no MRI abnormality was found. According to our study, we could rely on the predicting factors of persistent GH deficiency (abnormal MRI other than small anterior pituitary or Rathke pouch cyst, low GH peak and increased BMI) to determine which population could discontinue treatment at puberty. However, to confirm this strategy, a controlled prospective study is necessary. Many adolescents have poor adherence to follow-up. Paediatric endocrinologists must strive to improve the education provided to patients about the importance of continued multidisciplinary follow-up after adult height attainment. Moreover, due to loss of follow-up at the transitional phase many adult endocrinologists cannot explain to patients the importance and long-term benefits of treating adults with GHD. Special attention must be given to the transition from paediatric to adult care. Current recommendations indicate that GH provocative testing should be performed at least one month after GH therapy discontinuation. A single IGF-1 assay may be useful for screening: in our cohort, IGF-1 at adult height was significantly lower in patients with persistent GHD. However, the most strongly recommended test is the insulin tolerance test (ITT), which was used in all our patients. Alternatively, glucagon and arginine may be used in selected situations, with a BMI-dependent cut-off. Macimorelin is not yet widely available (10). However, macimorelin is approved only in patients over 18 years of age, thus not suitable for all patients during the transitional phase and no specific cut-off for macimorelin test in transition is available yet.

The main limitation of our study is its retrospective design. However, all patients were diagnosed and followed-up at the same highly specialized centre where both patient management and data recording were standardized.

In conclusion, in patients with non-tumoral isolated GHD, height <-2.5 SDS at diagnosis strongly predicted GHD persistence after adult height attainment. An abnormal pituitary region by MRI was the strongest single predictor. Over a quarter of patients had persistent GHD after linear growth completion, indicating the need to routinely test GH secretion after GH therapy discontinuation during the transition to adulthood. Continued GH therapy in adulthood in the event of persistent GHD is crucial to optimize the metabolic outcomes of these patients.

## Data availability statement

The original contributions presented in the study are included in the article/**Supplementary Material**. Further inquiries can be directed to the corresponding author.

## Ethics statement

The studies involving humans were approved by APHP ethics committee N° 2023 0202122136. The studies were conducted in accordance with the local legislation and institutional requirements. Written informed consent for participation in this study was provided by the participants’ legal guardians/next of kin.

## Author contributions

VM: Conceptualization, Investigation, Supervision, Validation, Writing – review & editing, Writing – original draft. GI: Writing – original draft, Methodology. GP: Visualization, Writing – review & editing. LG-B: Visualization, Writing – review & editing. DS-B: Visualization, Writing – review & editing. CT: Visualization, Writing – review & editing, Validation. IF: Visualization, Writing – review & editing. AS: Visualization, Writing – review & editing. JB: Visualization, Writing – review & editing. AB: Visualization, Writing – review & editing. SB: Visualization, Writing – review & editing. KG: Visualization, Writing – review & editing. ML: Visualization, Writing – review & editing. PT: Visualization, Writing – review & editing. GC: Visualization, Writing – review & editing, Methodology. MP: Visualization, Writing – review & editing, Conceptualization. DK: Conceptualization, Writing – review & editing, Investigation, Supervision, Validation.
