# Supplementary figures and images for: Identifying patient-related predictors of permanent growth hormone deficiency
Source: Front Endocrinol (Lausanne). 2023 Oct 10;14:1270845. doi: 10.3389/fendo.2023.1270845 (PMC10597646; doi:10.3389/fendo.2023.1270845)

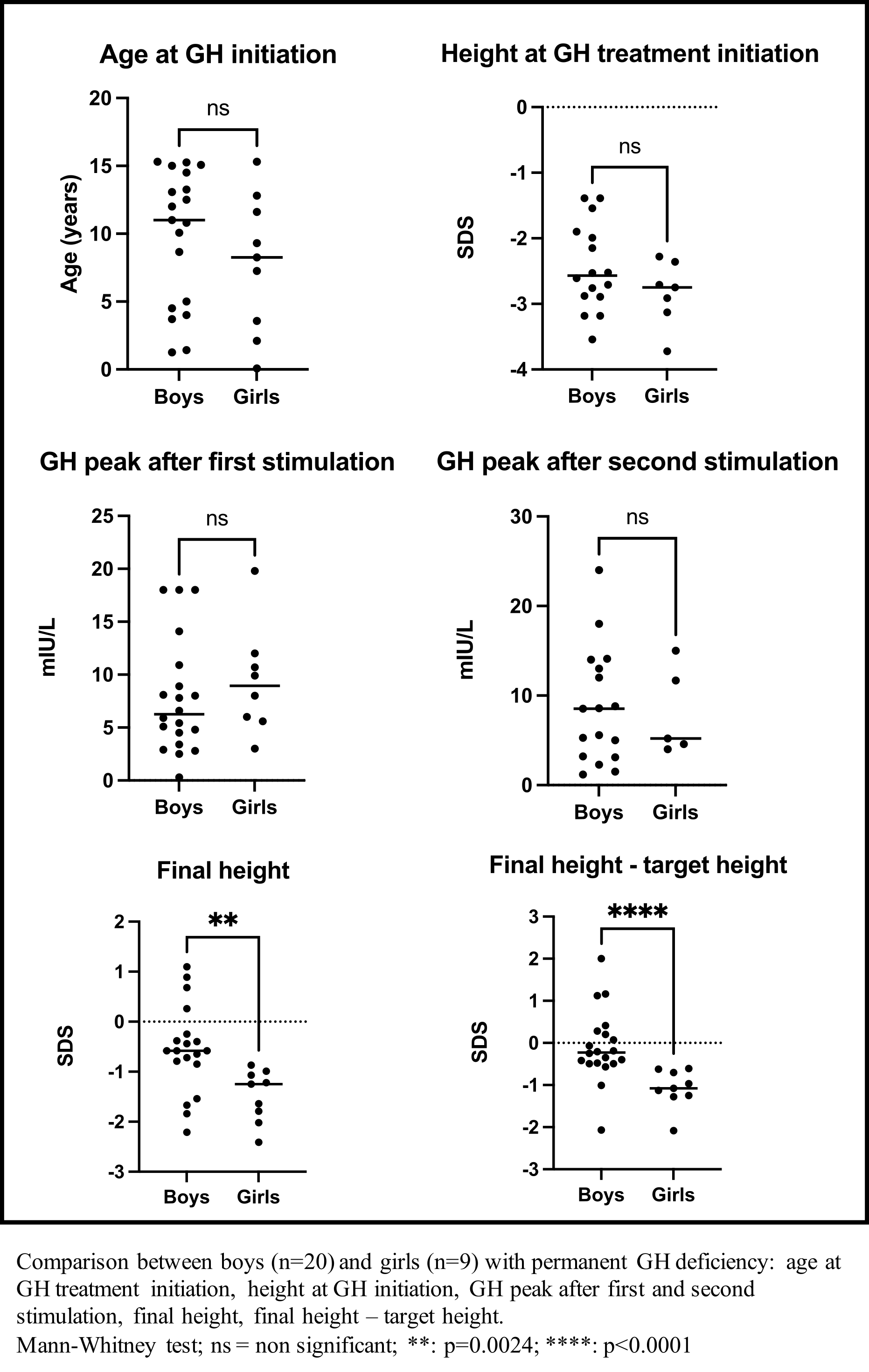

Supplement: Supplementary file 1 [file Image_1.tiff]

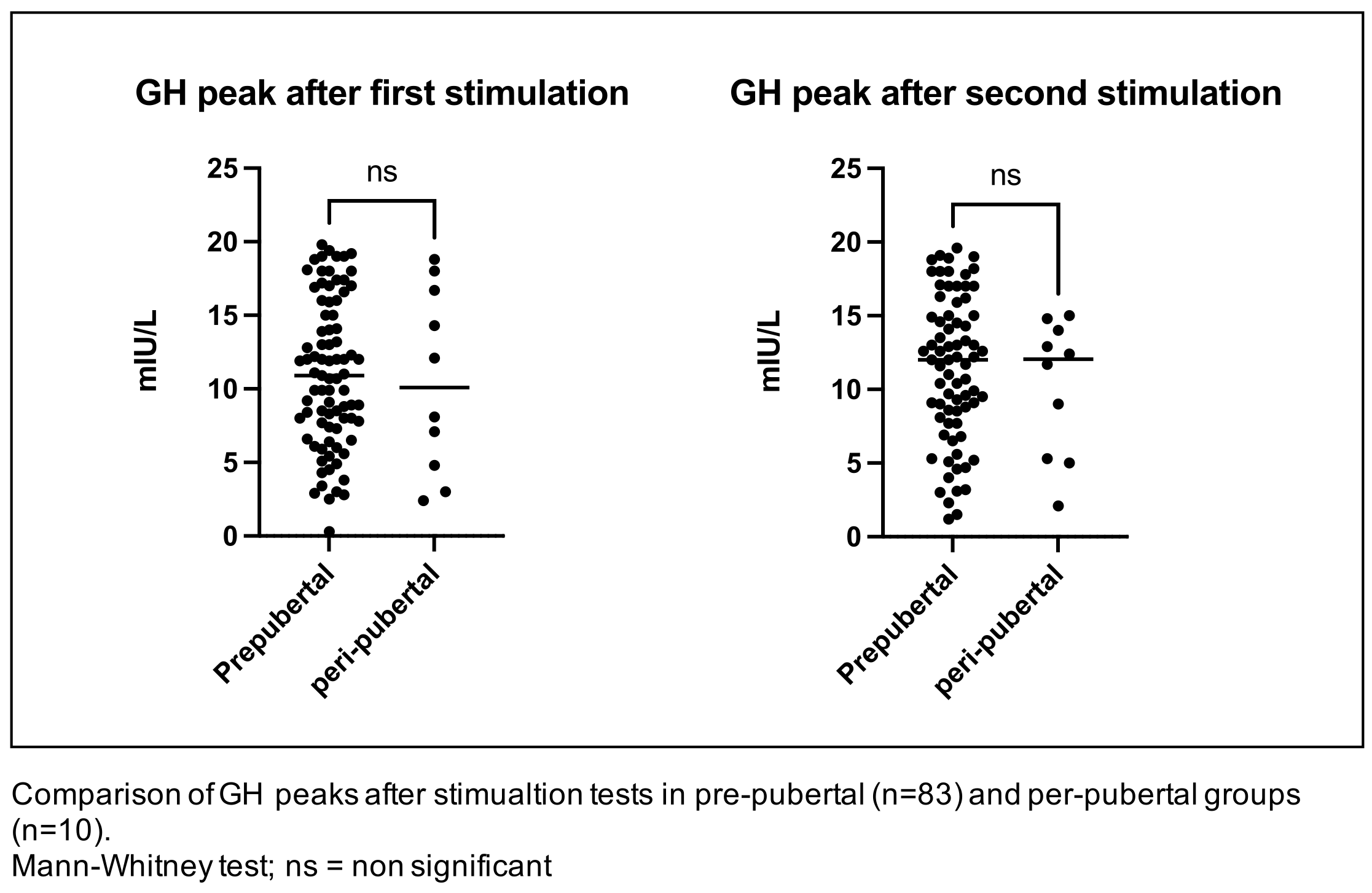

Supplement: Supplementary file 2 [file Image_2.tiff]
